# Association between adherence to Antiretroviral Therapy and place of residence among adult HIV infected patients in Ethiopia: A systematic review and meta-analysis

**DOI:** 10.1371/journal.pone.0256948

**Published:** 2021-09-02

**Authors:** Robera Olana Fite

**Affiliations:** HaSET Maternal and Child Health Research Program, Ethiopian Public Health Institute, Addis Ababa, Ethiopia; University of Sassari, ITALY

## Abstract

**Background:**

According to the World Health Organization, optimal adherence to Antiretroviral Therapy (ART) improves quality of life. Patients who use ART have varying characteristics in terms of where they live. The effect of place of residence on ART adherence is unclear in Ethiopia. Therefore, the aim of this systematic review and meta-analysis was to estimate the pooled association between place of residence and adherence to ART.

**Methods:**

Articles were retrieved from PubMed, Scopus, African Journals Online (AJOL), Journal Storage (JSTOR), and Web of Science. The data was extracted using Microsoft Excel 2016 spreadsheet. Review Manager 5.3 and STATA version 14 were used for the analysis. The Cochrane Q statistic was used to assess between-study heterogeneity. I^2^ was used to quantify between-study heterogeneity. A weighted inverse variance random-effects model was used to calculate the pooled odds ratio with 95% confidence interval.

**Results:**

Seven studies were included in this systematic review and meta-analysis. The Begg’s test (Z = 0.15, P = 0.881) and Egger’s test (t = 0.14, P = 0.894) revealed no evidence of publication bias. Urban residence was associated with an increased likelihood of good adherence (OR 2.07, 95%CI 1.22–3.51).

**Conclusions:**

The study recommends that policy-makers should enact policies that increase access to ART services in a rural area in order to improve adherence. It is recommended that implementation studies be conducted in order to identify practical and affordable interventions.

## Introduction

Acquired Immune Deficiency Syndrome (AIDS) is a viral disease caused by the Human Immunodeficiency Virus (HIV). Globally, 38 million people have acquired the disease. It is a major public health problem in developing countries [1]. The Sub-Saharan African countries were severely impacted by the HIV/AIDS pandemic [2]. In Ethiopia, 24,679 adult people are infected with HIV/AIDS [3].

A rapid and continuous response that includes both treatment and prevention activities is required to combat HIV/AIDS [4]. The treatment entails the administration of Antiretroviral therapy (ART) [5, 6]. ART services are provided for free in Ethiopia [4]. ART suppresses viral replication, which is an important component in extending life and improving quality of life in HIV reactive patients. It also helps to boost a patient’s immunity. This prevents HIV reactive patients from contracting opportunistic infections [7–9]. The goal of ART is to suppress HIV replication to the greatest extent possible. As a result, adherence is critical for a positive patient outcome. It also prevents the emergence of drug-resistant subspecies [10].

The World Health Organization (WHO) defines adherence as the extent to which a person’s behavior is consistent with the standardized recommendation made by the trained health professional. The behavior consists of taking medications, adhering to a diet, and/or implementing lifestyle changes [6].

Every patient interaction should include an assessment of ART adherence. Laboratory-based investigations and clinical evaluation should be conducted to assess drug tolerance and adverse drug effects [11]. Factors related to patients, health professionals, and health institutions all have an impact on adherence to ART [12, 13]. Patient-related factors that influence ART adherence include sex, age, residence, educational status, occupation, marital status, income, disclosure status, substance abuse history, clinical, and medication-related factors [12–16].

The patient, caregivers, and health professionals should work together throughout the ART provision period. Supportive psychological intervention must build a trusting relationship. Therefore, health professionals must be compassionate, respectful, and caring. This contributes to a higher level of adherence. This reduces the likelihood of treatment failure [11, 17–20].

Researchers in Ethiopia conducted a number of studies on adherence in both rural and urban areas of the country. HIV-positive patients live in both rural and urban areas. The effect of the study setting on the level of adherence requires an answer. To date, no systematic review or meta-analysis has been conducted in Ethiopia to estimate the association between place of residence and ART adherence. Therefore, the aim of this systematic review and meta-analysis is to answer the question of whether there is an association between the place of residence and good adherence to ART by estimating the pooled effect of place of residence on adherence to ART among adult HIV-infected patients in Ethiopia. The findings will help policymakers focus on the effect of residence on ART adherence and develop strategies to improve service accessibility for patients living in rural areas.

## Methods

### Study design and search strategy

This systematic review and meta-analysis was conducted to estimate the association between ART adherence and place of residence. A systematic search for articles was conducted in PubMed, Scopus, African Journals Online (AJOL), Journal Storage (JSTOR), and Web of Science. The author searched for articles written in English. After consulting with an expert and a librarian, the electronic databases were chosen.

The core search terms and phrases were: “HIV”, “AIDS”, “HIV infected”, “HIV/AIDS”, “Acquired Immune Deficiency Syndrome”, “Human Immunodeficiency Virus”, “Adherence”, “Non-adherence”, “Compliance”, “Non-compliance”, “Antiretroviral”, “Treatment Adherence”, “Failure to Adhere”, Antiretroviral Therapy”, “ART”, “HAART”, “Highly Active Antiretroviral Therapy”, “Combined Antiretroviral”, “Viral suppression”, “Pill Count”, “Ethiopia”, “Addis Ababa”, “Harar”, “Dire Dawa”, “SNNP”, “Oromia”, “Amhara”, “Somali”, “Tigray”, “Gambella”, “Benishangul Gumuz”, “Afar”.

The following terms with MeSH (Medical Subject Headings) and Boolean operators were used to search PubMed:

((((((((((((((((((((HIV) OR (("HIV Long-Term Survivors"[Mesh] OR "Anti-HIV Agents"[Mesh]) OR ("HIV Infections"[Mesh] OR "HIV Seroprevalence"[Mesh]))) OR Human Immunodeficiency Virus) OR AIDS) OR ("Acquired Immunodeficiency Syndrome"[Mesh] OR "AIDS-Related Opportunistic Infections"[Mesh])) OR HIV/AIDS) AND Adherence) OR ("Treatment Adherence and Compliance"[Mesh] OR "Medication Adherence"[Mesh] OR "Patient Compliance"[Mesh])) OR Compliance) OR Non-adherence) OR Non-compliance) OR Pill count) AND Antiretroviral) OR ("Anti-Retroviral Agents"[Mesh] OR "Antiretroviral Therapy, Highly Active"[Mesh])) OR Antiretroviral Therapy) OR ART) OR HAART) AND Residence) OR "Residence Characteristics"[Mesh]) AND Associated factors) AND Ethiopia

The findings of this systematic review and meta-analysis have been reported in accordance with the Preferred Reporting Items for Systematic Review and Meta-Analysis (PRISMA) guideline [21] (S1 Checklist).

### Study selection and eligibility criteria

The inclusion criteria were:

#### Study period

This study included studies completed or published up to March 30, 2020.

#### Study type

This study included all observational studies that reported on ART adherence and associated factors.

#### Language

This study included studies that were published in English.

#### Population

This study included studies that were conducted on HIV-infected patients.

#### Place of study

The study included studies conducted in Ethiopia.

#### Publication status

This study included both published and unpublished articles.

#### Content of study

This study included articles that identified adherence to ART and associated factors.

Articles were excluded if they were either review articles or studies that did not report the desired outcome.

### Study selection

The author exported the retrieved studies to EndNote X7, which was then used to eliminate duplicate studies. The author determined the eligibility of the candidate studies. The abstract title and content were used to screen the articles. The screened articles were then subjected to a full article review. The inclusion and exclusion criteria were used to screen the articles.

### Quality appraisal

The quality of the studies included in the systematic review and meta-analysis was assessed using the Joanna Briggs Institute (JBI) quality appraisal checklist. The appraisal checklist included the following parameters: 1) inclusion criteria, 2) description of study subject and setting, 3) valid and reliable measurement of exposure, 4) objective and standard criteria used, 5) identification of confounder, 6) strategies to handle confounder, 7) outcome measurement, and 8) appropriate statistical analysis. If the quality assessment indicator score was 50% or higher, the study was considered low risk. All of the articles included in this systematic review and meta-analysis were found to be low risk.

### Data extraction

A standardized data extraction format was carefully designed in a Microsoft Excel 2016 spreadsheet that was used to extract data from the articles. The first author’s name, publication year, study period, study setting, region, study design, sample size, sampling method, adherence definition, measures, and adherence level in both urban and rural areas were all extracted.

### Study measures

This study estimated the pooled association between place of residence and adherence. The residence is a dichotomous variable that can be classified as urban or rural. Adherence is another dichotomous variable that can be classified as good or bad. Table 1 shows the studies’ definition of good adherence.

**Table 1:** Characteristics of the study involved in the systematic review and meta-analysis.

Author	Publication Year	Study period	Study Setting	Region	Study design	Sampling method	Sample Size	Measures	Adherence definition	Good Vs. Bad Adherence		Quality
										Urban	Rural	
Legesse TA et al. [24]	2019	April-May 2017	Hara Health Center	North-Eastern Ethiopia	Cross-Sectional	NR	418	Patient-self report	Take ≥95% (<2 doses of 30 doses or <3 doses of 60 doses are missed)	217/61	83/57	Low risk
Molla AA et al. [23]	2018	May-June 2015	University of Gondar Referral Hospital	Amhara	Cross-Sectional	Systematic random sampling	440	Patient-self report	Take ≥95% of the prescribed medication 1 month prior to survey	347/30	41/22	Low risk
Chaka TE et al. [26]	2016	No data	Nejo, Gimbi, Adama, Weji, Negele, Gelemso, Negele Hospitals and Nekemte, Wolenchiti, Batu, Mojo, Shakiso Health centers	Oromia	Cross-Sectional	Consecutive sampling	1631	Logbook and patient interview	Take more than 95% of the prescribed doses correctly one month prior to the survey	1310/32	276/13	Low risk
Bitew BD et al. [27]	2014	December 5 /2013- February 10/2014	Arba Minch General Hospital	SNNP	Unmatched Case-control	NR	Cases-115	Patient self-report	Took ≥ 95% (missing ≤2 doses of 30 doses or ≤3 doses of 60 doses)	300/75	47/40	Low risk
							Controls-347					
Abera A et al. [28]	2015	February-March 2015	Jimma University Teaching Hospital,	Oromia	Cross-Sectional	Convenience Sampling	221	NR	Good adherence: Those who took 95% or more of the drug prescribed	51/116	29/25	Low risk
Hailasillassie K et al. [25]	2014	June to July 2012	Mekelle Hospital	Tigray	Cross-Sectional	Systemic random sampling	403	Patient Interview	Correctly answer eight items Morisky medication adherence scale	233/128	18/24	Low risk
Tegegne AS et al. [22]	2018		Felege Hiwot Teaching and Specialized Hospital	North-western Ethiopia	Prospective study	Random sampling	792	Pill count	Take ≥95% of the prescribed pills	150/228	114/300	Low risk

### Statistical analysis

Pooled analysis was done using Review Manager 5.3(Version 5.3. Copenhagen: The Nordic Cochrane Centre, The Cochrane Collaboration, 2014). Publication bias was assessed using STATA 14 software (stataCorp LP, 4905 Lakeway Drive, College Station, TX 77845, USA). The number of adherent and non-adherent HIV-infected patients in urban and rural areas is entered to calculate the odds ratios (OR). The Cochrane Q statistic was used to determine whether there was significant between-study heterogeneity. I^**2**^ was used to quantify between-study heterogeneity, with values of 0%, 25%, 50%, and 75% representing no, low, medium, and increased heterogeneity, respectively. Because of the observed heterogeneity between the studies, a weighted inverse variance random-effects model was used to calculate the pooled odds ratio with a 95% confidence interval.

The publication bias was checked using Begg’s rank correlation test and Egger’s linear regression test. Publication bias is not the only source of asymmetry in funnel plots. In addition, the visual inspection of funnel plots is subjective and should be supplemented with additional analysis. Therefore, the funnel plot was used to examine small-study effects. Begg’s rank correlation test and Egger’s linear regression test were used by the author because the tests had stronger discriminatory power in detecting publication bias than Macaskill’s method. Furthermore, Deeks’ method was not used because it is originally designed for meta-analysis of diagnostic tests.

A p-value of 0.05 was used in this systematic review and meta-analysis to determine the significance of the small study effect. The absence of publication bias was demonstrated by Begg’s rank correlation test (Z = 0.15, P = 0.881) and Egger’s linear regression test (t = 0.14, P = 0.894).

## Results

**Fig 1:**
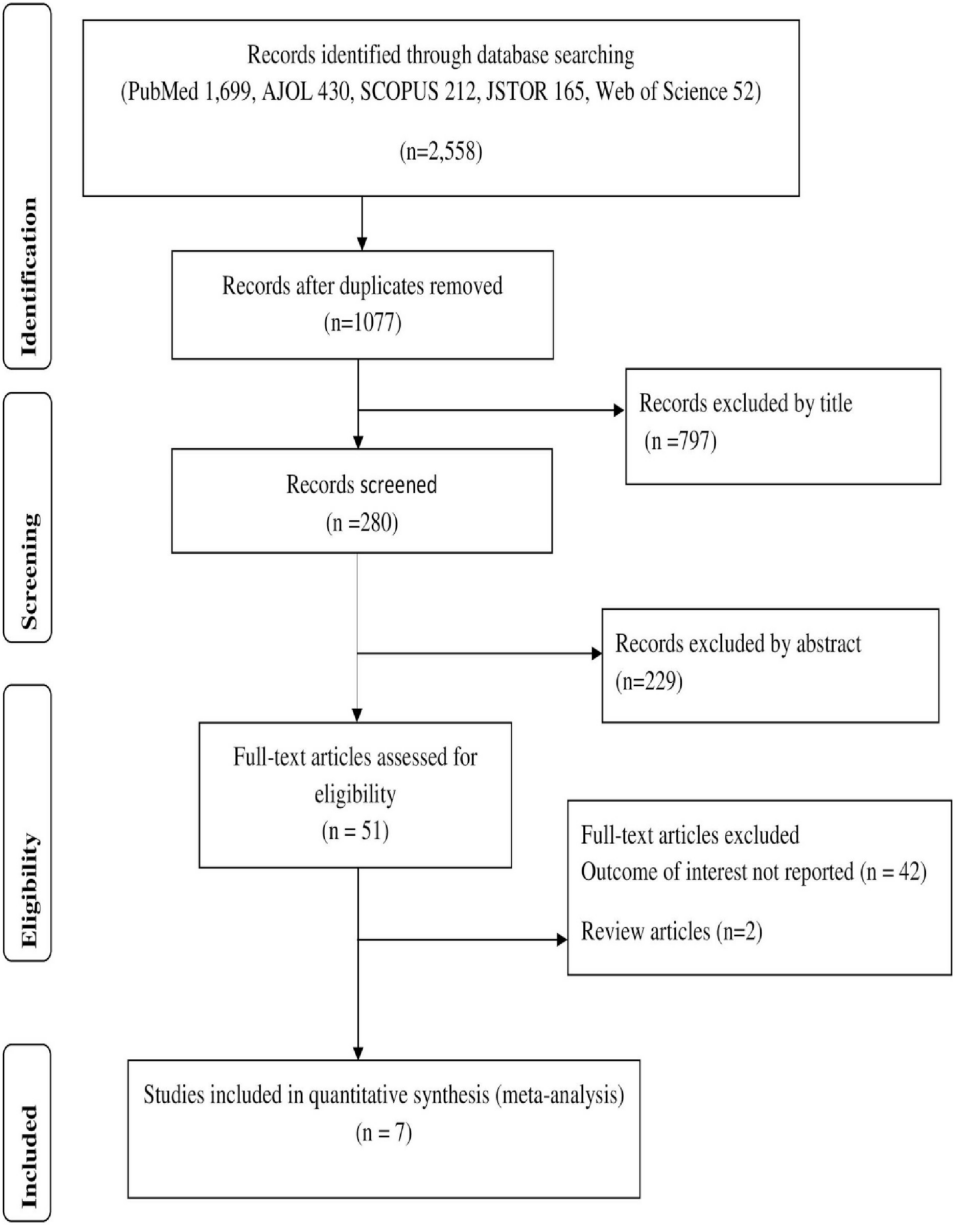
Flow chart of study selection.

Initially a total of 2558 articles were retrieved: 1699 from PubMed, 430 from AJOL, 212 from SCOPUS, 165 from JSTOR, and 165 from Web of Science databases. After duplicates were removed, 1077 remained. Two hundred articles were screened based on their abstracts. Of these, 51 articles were assessed using the eligibility criteria. Finally, 7 articles met the eligibility criteria and were included in the quantitative synthesis (Fig 1).

### Study characteristic

The studies’ publication years’ ranged from 2014 to 2019 [22–28]. All the articles involved in this systematic review and meta-analysis showed a low risk. Five studies were conducted in hospitals [22, 23, 25, 27, 28] and one study was conducted in a health center [24]. The other one study was conducted in both hospitals and health centers [26]. Six studies used a cross-sectional study design [22–26, 28], and one study used a case-control study design [27]. One study was conducted in Northeastern Ethiopia [24], one study was conducted in Amhara region [23], two studies were conducted in Oromia region [26, 28],one study was conducted in Northwestern Ethiopia [22], one study was conducted in Southern Nations, Nationalities and Peoples (SNNP) [27], and one study was conducted in Tigray [25]. The actual sample size ranged from 221 to 1631 [22–28]. Two studies used the systematic random sampling method [23, 25], and one study used the random sampling method [22]. One study selected study participants consecutively [26], while another study selected study participants conveniently [28]. Six studies used an adherence threshold of 95% or higher as a cut point to define good ART adherence [22–24, 26–28]. One study used the Morisky medication adherence scale [25] (Table 1).

### Association between place of residence and adherence

**Fig 2:**
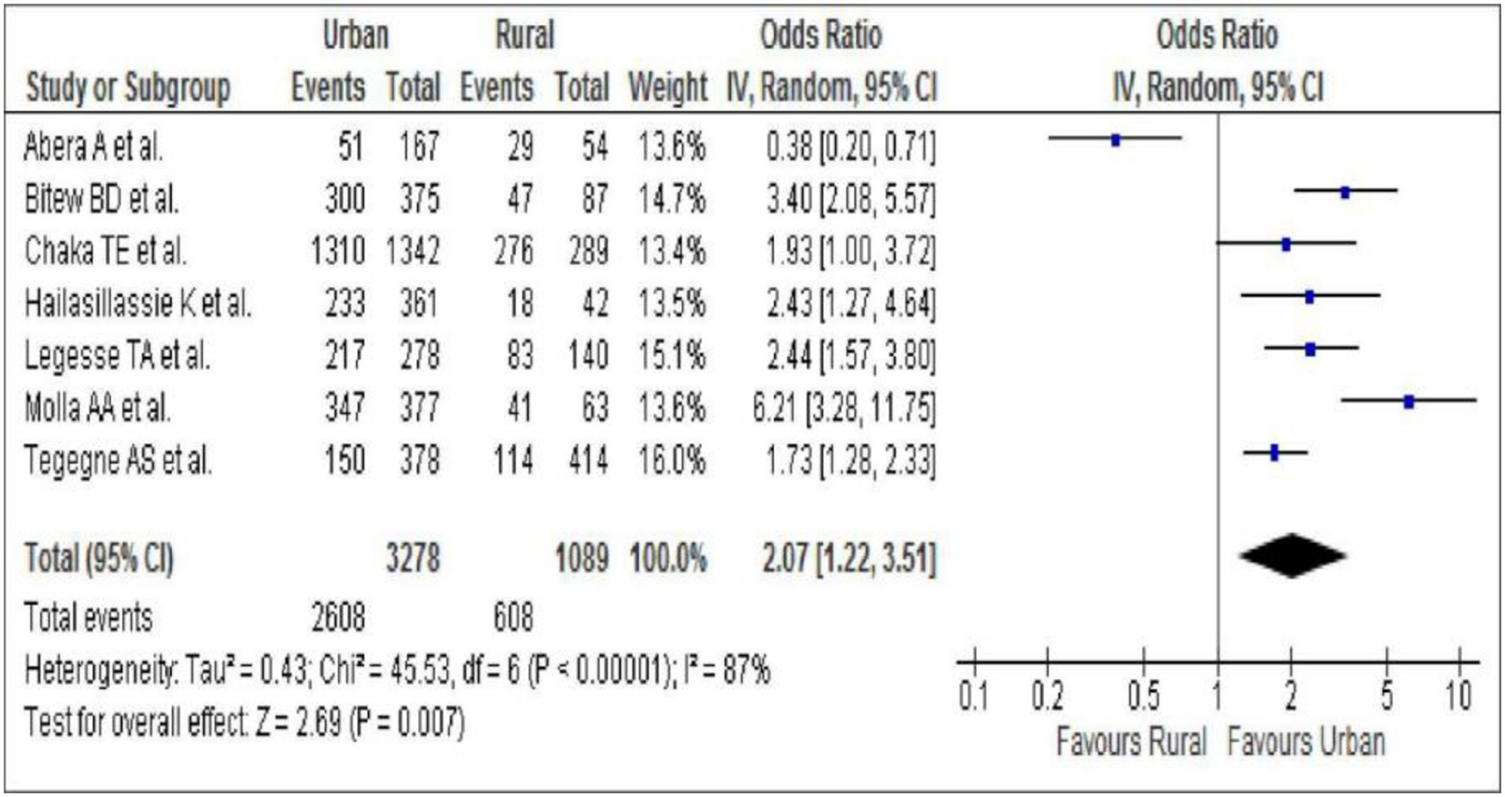
Pooled association between ART adherence and place of residence.

The pooled association between ART adherence and residence was estimated using seven studies. The OR was used to estimate the association. The OR ranges from 0.38(95%CI 0.20–0.71) to 6.21(95%CI 3.28–11.7). The result showed a high level of heterogeneity (Heterogeneity chi-square 45.54, df. 6, I^2^ 87%, P<0.001). Therefore, the pooled association between ART adherence and residence was determined using a random-effects model. Increased likelihood of good ART adherence was identified among patients living in urban areas. Accordingly, HIV-infected patients living in urban areas were 2.07 times more likely to have good ART adherence as compared to HIV-infected patients living in rural areas (OR 2.07, 95%CI 1.22–3.51) at p = 0.007 (Fig 2).

### Publication bias

**Fig 3:**
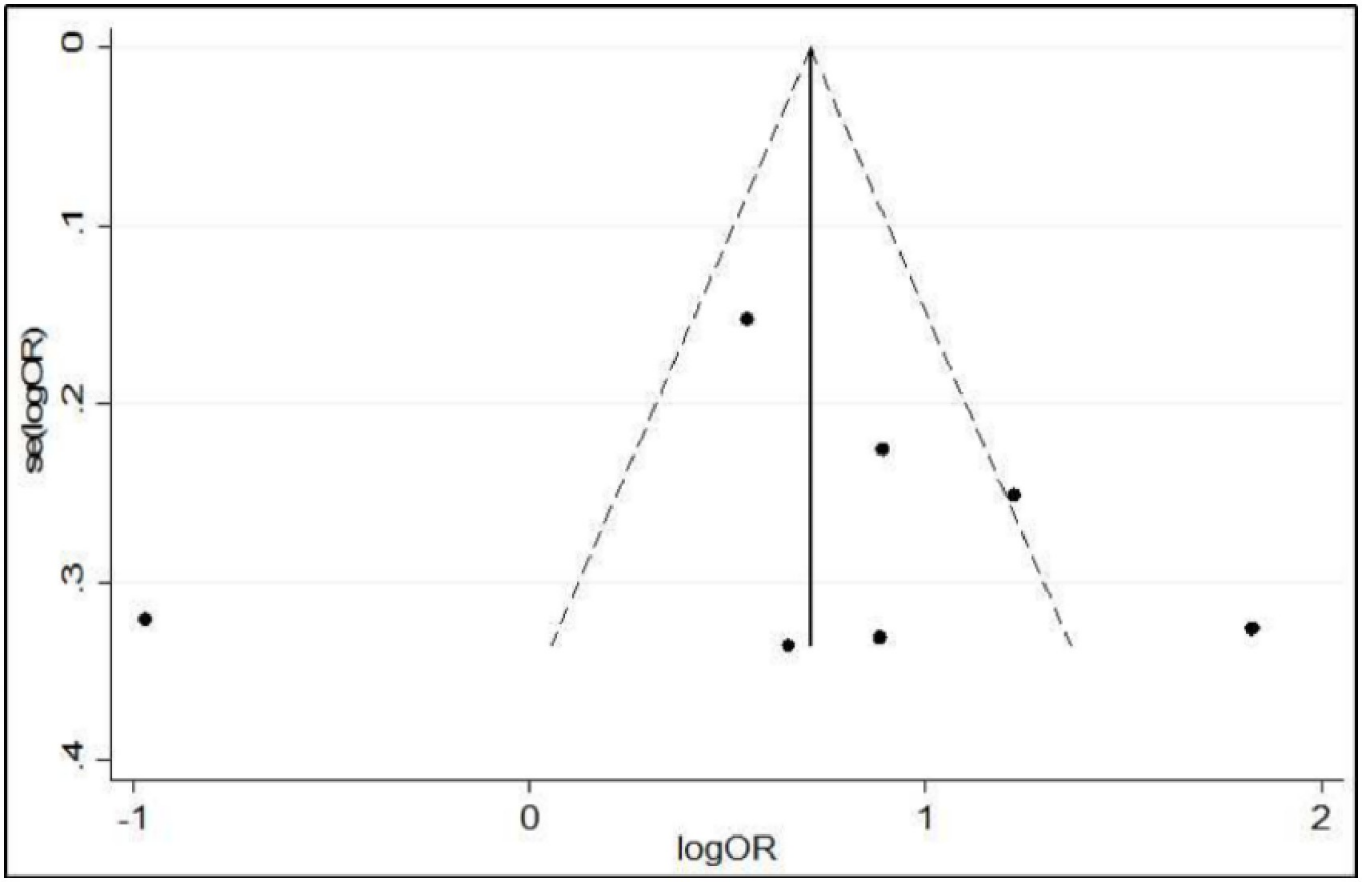
Funnel plot of publication bias.

The funnel plot revealed a symmetrical distribution (Fig 3). The Egger’s linear regression test and Begg’s rank correlation test were used to objectively identify publication bias. Egger’s linear regression test was not statistically significant (t = 0.14, P = 0.894). Furthermore, Begg’s rank correlation test was not statistically significant (z = 0.15, P = 0.881) (Table 2). Therefore, Begg’s rank correlation test and Egger’s linear regression test revealed no evidence of publication bias.

**Table 2:** Egger’s test for small-study effects.

Standard Effect	Coefficient	Standard Error	t	P>|t|	95% CI
**Slope**	0.5865918	0.9362655	0.63	0.558	-1.820155,2.993339
**Bias**	0.5319097	3.811847	0.14	0.894	-9.266754,10.33057

## Discussion

Seven studies were included in this systematic review and meta-analysis to summarize the effect of place of residence on the level of ART adherence among adult HIV-infected patients living in Ethiopia. The findings were compiled from studies conducted in various regions of Ethiopia by the author.

Adherence to ART is related to the quality of life of HIV-infected patients [29]. Patients in developing countries are expected to overcome the challenges of the health care system in order to achieve a high quality of life. This problem is exacerbated by a lack of access to ART services in nearby health institutions [8, 9, 16].

The finding showed that patients living in urban areas are 2.07 times more likely to have a good adherence as compared to those living in rural areas. This finding is supported by a finding from a study conducted in Kenya, which analyzed the results of the second Kenya AIDS indicator survey [30]. It is also supported by a systematic review and meta-analysis, which reported that patients living in urban areas are more likely to have a good adherence as compared to those living in rural areas [31]. This finding is supported by another systematic review conducted in South Africa on equity in utilization of ART [32]. On the other hand, the finding is contrary to a report from a systematic review conducted in Sub-Saharan African countries, which showed that urban residence favors non-adherence [16]. The poor ART adherence among patients living in rural areas could be attributed to a variety of factors. People in Ethiopia’s rural areas have a low socioeconomic status. Likewise, HIV-infected patients living in rural areas have a low income. This might lead to poor adherence. In addition, poor adherence is linked to a lack of access to ART services [33]. HIV-infected patients in urban areas who live close to the health facility use of the service frequently. In Ethiopia, most health facilities are not located in rural areas, and patients are expected to travel to urban areas to receive ART services. Therefore, they may miss an appointment and exhibit poor adherence. The presence of a support system is also related to adherence [34]. Patients in rural areas may have a deficient support system.

Asymmetrical funnel plots may indicate publication bias or exaggeration of treatment effects in small, low quality studies. Therefore, the author used Begg’s rank correlation test and Egger’s linear regression test to check publication bias. The results of the statistical tests showed there was no publication bias.

One of the studies included in this systematic review and meta-analysis used a consecutive sampling method to select study participants and didn’t take into account the sampling bias introduced by this method [26]. Studies that collected data using self-reporting did not consider self-reporting bias, social desirability bias, and recall bias [23–25, 27]. It is possible that it will have an effect on the reported data from the studies. In addition, one of the studies conducted at Jimma University Teaching Hospital selected study participants using convenience sampling, which is a type of non-probability sampling [28]. It is possible that it will have an impact on the study’s findings because it is influenced by sampling error and selection bias.

Each article was subjected to a quality assessment before being included in the systematic review and meta-analysis in order to improve the validity of the results. Adherence was measured using a standard method in the articles involved in this study. In addition, publication bias was checked because it affects the validity of the results. In selecting the appropriate database, the author also consulted an expert and a librarian.

The study recommends that policymakers focus on incorporating ART services into rural health centers and health posts. A more stringent engagement strategy should be developed to encourage the involvement of sectors working to combat HIV/AIDS. The health institutions that provide ART services should have a mechanism in place to monitor patient adherence. The facilities could collaborate with health extension workers in the rural area to monitor the patient. Adherence could be improved through a close monitoring and follow-up. Health facilities can also launch a special program that serves as a platform to thoroughly discuss the patient-perceived barriers. The patient should also communicate openly with the health professionals who provide ART services, which can make it easier for health professionals and patients to overcome barriers.

This systematic review and meta-analysis is not free from limitations. These included the author’s inability to determine the temporal relation due to the study designs used in the articles. Furthermore, the studies were not conducted across the country. The lack of multiple studies in various regions of the country makes it difficult to conduct a subgroup analysis. Therefore, the factors contributing to heterogeneity were not identified. Since the studies included in this systematic review and meta-analysis had small sample sizes, the findings should be interpreted with caution.

## Conclusions

The results of this systematic review and meta-analysis showed a significant association between place of residence and adherence to ART. Accordingly, living in urban areas was significantly associated with an increased likelihood of good ART adherence. Living in rural areas, on the other hand, reduces the likelihood of good adherence. Therefore, all-inclusive and rigorous discussions should be organized at the national level on mechanisms to improve adherence in HIV-infected patients living in rural areas. Furthermore, national-level studies should be conducted to identify the reasons for the effect of the place of residence on ART adherence. Implementation researches that could identify practical and affordable interventions are recommended.

## Supporting information

S1 Checklist PRISMA checklist. (DOC)
